# First detailed reconstruction of the karyotype of Trachypithecus cristatus (Mammalia: Cercopithecidae)

**DOI:** 10.1186/1755-8166-6-58

**Published:** 2013-12-17

**Authors:** Fan Xiaobo, Krit Pinthong, Hasmik Mkrtchyan, Pornnarong Siripiyasing, Nadezda Kosyakova, Weerayuth Supiwong, Alongkoad Tanomtong, Arunrat Chaveerach, Thomas Liehr, Marcelo de Bello Cioffi, Anja Weise

**Affiliations:** 1 Institute of Human Genetics, Jena University Hospital, Friedrich Schiller University, Kollegiengasse 10, Jena D-07743, Germany; 2 Faculty of Science and Technology, Surindra Rajabhat University, 186 Moo 1, Surin, Maung District 32000, Thailand; 3 Center of Medical Genetics and Primary Health Care, Abovyan Str 34/3, 001, Yerevan, Armenia; 4 Faculty of Science and Technology, Rajabhat Maha Sarakham University, 80 Nakonsawan Rd, Maha Sarakham, Talad, Maung District 44000, Thailand; 5 Department of Biology, Faculty of Science, Khon Kaen University, 123 Moo 16 Mittapap Rd., Khon Kaen, Muang District 40002, Thailand; 6 Departamento de Genética e Evolução, Universidade Federal de São Carlos, São Carlos, SP, Brazil; 7 Institut für Humangenetik, Postfach, Jena D-07740, Germany

**Keywords:** Evolutionary conserved breakpoints, Multicolor banding, Old world monkeys, XY_1_Y_2_ sex system

## Abstract

**Background:**

The chromosomal homologies of human (Homo sapiens = HSA) and silvered leaf monkey (*Trachypithecus cristatus* = TCR) have been previously studied by classical chromosome staining and by fluorescence in situ hybridization (FISH) applying chromosome-specific DNA probes of all human chromosomes in the 1980s and 1990s, respectively.

**Results:**

However, as the resolution of these techniques is limited we used multicolor banding (MCB) at an ~250-band level, and other selected human DNA probes to establish a detailed chromosomal map of TCR. Therefore it was possible to precisely determine evolutionary conserved breakpoints, orientation of segments and distribution of specific regions in TCR compared to HSA. Overall, 69 evolutionary conserved breakpoints including chromosomal segments, which failed to be resolved in previous reports, were exactly identified and characterized.

**Conclusions:**

This work also represents the first molecular cytogenetic one characterizing a multiple sex chromosome system with a male karyotype 44,XY_1_Y_2_. The obtained results are compared to other available data for old world monkeys and drawbacks in hominoid evolution are discussed.

## Background

*Trachypithecus cristatus* (TCR) [1], also known as silvered lutung, silvered leaf monkey or the silvery langur, belongs to superfamily Cercopithecoidea, family Cercopithecidae, subfamily Colobinae. The colobines divided during evolution into an African clade and an Asian clade [2]. TCR is widely distributed in continental Southeast Asia including Myanmar, West-central Thailand, Cambodia, Laos, Vietnam and Southern China [2-4]. Recently, four species groups were recognized (*T. pileatus*, *T. francoisi*, *T. obscurus* and *T. cristatus*), including 18 species only in the Asian colobine of *Trachypithecus*. Genus TCR was initially denominated with various Latin names between 1821 and 1962, like *Simia cristata*, *Semnopithecus cristata*, *Pygathrix cristata, Presbytis cristata*, before the current name *Trachypithecus cristatus* was introduced [2].

The karyotype of TCR was described in 1970 as 2n = 44 [5]. In the 1980s, chromosome banding analysis were used in TCR [6-8], including comparative R-banding of three different species of *Colobus* genus [6]. In 1983, G and Q banding were applied to analyze the banding patterns of a female TCR [7]. One year later, male TCR was characterized as carrying an evolutionary conserved translocation involving the Y chromosome and two autosomes [9]. Furthermore, since 1997 chromosomal homologies between human chromosomes and TCR has been established by fluorescence in situ hybridization (FISH) applying human whole chromosome paintings. Thus, up to now, unique reciprocal translocations corresponding to HSA Y & 5, HSA 1 & 19, and HSA 6 & 16 as well as fusions of HSA 14 & 15 and HSA 21 & 22, were characterized [10]. However, as whole chromosome paints have only a limited resolution [11], we established a detailed comparative chromosome map of TCR primarily based on multicolor banding (MCB). The potential of this approach in order to clarify and to resolve evolutionary conserved chromosomal rearrangements was already shown by our group for other primates [12-14].

## Results

**Figure 1:**
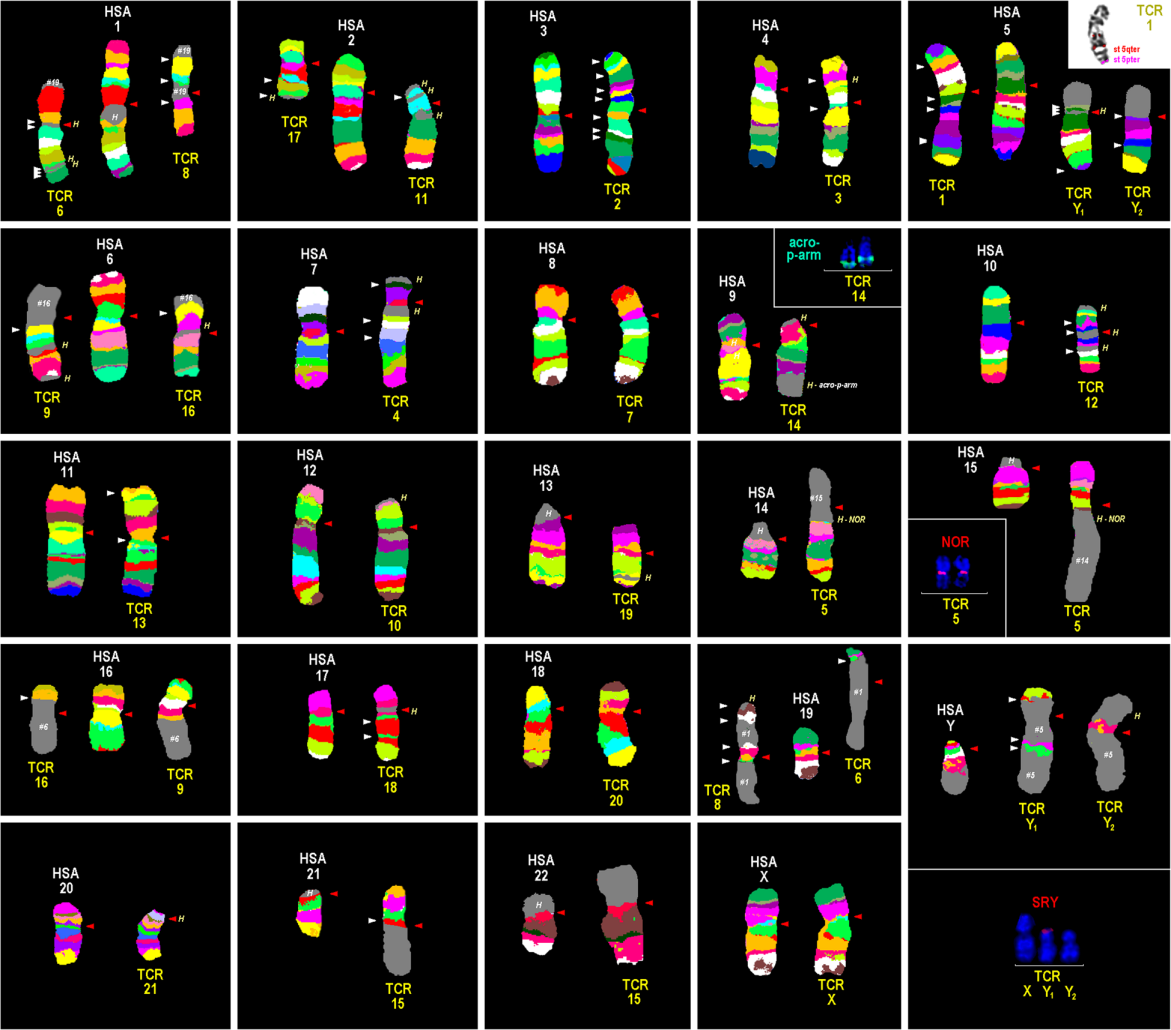
**Representative results from this study using human MCB probes on TCR in comparison to HSA chromosomes are depicted as pseudo-colored results of HSA and TCR, at an ~250-band level.** HSA chromosomes are numbered by white figures, TCR chromosomes by yellow figures. The chromosomes are sorted here according to the HSA-chromosomes. In subfigures for HSA 5, 9, 14 and Y there are additional FISH-results shown using probes as indicated. Red arrows stand for TCR centromeres and white arrows for HSA-centromeres. Abbreviations: # = number of a human chromosome – region not stained by MCB probe; acro-p-arm = probe for all acrocentric short arms in HSA; H = heterochromatin, HSA = *Homo sapiens*; NOR = HSA specific FISH-probe for nucleolus organizing region; st = subtelomere; SRY = HSA specific FISH-probe for sex determining region Y; TCR = *Trachypithecus cristatus*.

MCB results are summarized in Figure 1 and in Tables 1 and 2. Representative results of probes specific for all acrocentric short arms in HSA, the NOR-region and SRY are also shown in Figure 1. As outlined in Table 1 the majority of TCR chromosomes are completely homologous to one of the human chromosomes, exceptions are only TCR 5 (homologous to HSA 14 and 15), TCR 6 and 8 (homologous to HSA 1 and 19), TCR 9 and 16 (homologous to HSA 6 and 16), TCR 15 (homologous to HSA 21 and 22), and TCR Y1 and Y2 (homologous to HSA Y and 5).

**Table 1:** Homologous regions, centromere position and heterochromatic inserts observed in this study of TCR compared to HSA chromosomes

**TCR chromosome**	**Homologous HSA-regions (for rearrangements see Table**2**)**	**Centromere position**	**Heterochromatic inserts in**
TCR 1	HSA 5pter-5qter	like in HSA 5	n.d.i.t.s.
TCR 2	HSA 3pter-3qter	HSA 3q26	n.d.i.t.s.
TCR 3	HSA 4pter-4qter	like in HSA 4	n.d.i.t.s.
TCR 4	HSA 7pter-7qter	like in HSA 7	- end of HSA 7p
			- 7q11.1
TCR 5	HSA 15q11.2-15qter, HSA 14q11.2-14qter	HSA 15q26.1 ~ 26.2	- fus HSA 14q11.2 / 15q26.3
TCR 6	HSA 1p22-1qter, HSA 19pter-19p13.2	fus HSA 1q22 / 1p14	- fus HSA 1q22 / 1q41;
			- fus HSA 1q41 / 1p22;
			- 1q24
TCR 7	HSA 8pter-8qter	like in HSA 8	n.d.i.t.s.
TCR 8	HSA 1pter-1p22, HSA 19p13.2-19qter	like in HSA 19	- end of HSA 19q
TCR 9	HSA 6pter-6q15, HSA 16p13.1-16qter	like in HSA 16	- end of HSA 6p;
			- 6p21
TCR 10	HSA 12pter-12qter	like in HSA 12	- end of HSA 12p
TCR 11	HSA 2q14.1-2qter	HSA 2q24.2	- distal from HSA 2q14.1;
			- 2q21;
			- 2q31
TCR 12	HSA 10pter-10qter	like in HSA 10	- end of HSA 10p;
			- fus HSA 10q21.1 / 10q22.3
			- HSA 10p11.1
			- HSA 10q11.1
TCR 13	HSA 11pter-11qter	HSA 11p15.3	n.d.i.t.s.
TCR 14	HSA 9pter-9qter	HSA 9q33 ~ 34.1	- end of HSA 9q
			- end of HSA 9p
TCR 15	HSA 21q11.2-21qter, HSA 22q11.21-22qter	fus 21q11.2 / 22q11.21	n.d.i.t.s.
TCR 16	HSA 6q15-6qter, 16pter-16p13.1	HSA 6q21	- HSA 6q21
TCR 17	HSA 2pter-2q14.1	like in HSA 2	- end of HSA 2p
TCR 18	HSA 17pter-17qter	like in HSA 17	- HSA 17p11.1
TCR 19	HSA 13q12.1-13qter	HSA 13q14	- HSA 13q32 ~ 33
TCR 20	HSA 18pter-18qter	HSA 18q21	n.d.i.t.s.
TCR 21	HSA 20pter-20qter	HSA 20p12	- HSA 20p11.1
			- HSA 20q11.1
TCR X	HSA Xpter-Xqter	like in HSA X	n.d.i.t.s.
TCR Y_1_	HSA 5p12-5q31.2, HSA Ypter-Yp11.2	like in HSA 5	- HSA 5p11
			- HSA 5q11.1
TCR Y_2_	HSA 5pter-5p12, HSA 5q31.2-5qter,HSA Yp11.2-Yq11.23	like in HSA Y	- distal from HSA Yp11.23

*Abbreviations:* fus = fusion of; n.d.i.t.s. = none detected in this study.

**Table 2:** Evolutionary conserved breakpoints in TCR chromosomes compared to HSA; the positions are analyzed concerning their location in GTG-light bands, colocalization with human fragile sites and breakpoints observed in HLA and GGO using MCB-approach

**HSA chr.**	**Evolutionary conserved breakpoints including neo-centromere and heterochromatin positions**	**GTG- light band**	**Fragile site in same band**	**Same breakpoint in HLA [Mrasek et al., 2003 **[13]**]**	**Same breakpoint in GGO [Mrasek et al., 2001 **[12]**]**
1	1p22	+	FRA1D	-	-
	1q22	-	-	-	-
	1q24	-	-	+	-
	1q41	-	FRA1R	-	-
2	2p25.3	-	FRA2M	-	-
	2q14.1	-	FRA2	-	+
	2q21	+	FRA2F	-	?+
	2q24.2	+	FRA2T	+	-
	2q31	+	FRA2G	-	-
3	3p26.3	-	FRA3E	-	-
	3p25	+	FRA3F	-	-
	3p23	+	-	-	-
	3p21.3	+	FRA3H	?+	-
	3p14	-	FRA3B	?+	-
	3q22	-	FRA3N	?+	-
	3q25	+	FRA3D	+	-
	3q26	-	FRA3O	-	-
	3q28	-	FRA3P	-	-
4	4p12	+	FRA4H	-	+
	4q22	-	FRA4F	-	-
5	5p15.2	-	FRA5H	-	-
	5q11.2	+	FRA5I	?+	-
	5q21	-	FRA5F	-	-
	5q31.2	-	FRA5C	-	-
	5q35.3 (a)	+	FRA5G	-	+
	5q35.3 (b)	+	FRA5G	-	+
6	6p25.3	+	-	-	-
	6p21	+	FRA6H	-	-
	6q15	+	FRA6G	+	-
	6q21	+	FRA6F	-	-
7	7p22.3	+	FRA7B	+	-
	7p15.3	+	-	-	-
	7q11.1	-	FRA7A	-	-
	7q11.23	+	FRA7J	-	+
9	9p34.2	-	-	-	-
	9q24.3	+	-	-	-
	9q33 ~ 34.1	-	FRA9M	-	-
10	10p15.3	+	FRA10H	-	-
	10p11.2	+	FRA10J	-	?+
	10p11.1	-	-	-	-
	10q11.1	-	FRA10G	-	-
	10q21.1	-	FRA10C	+	-
	10q22.3	+	FRA10D	?+	+
11	11p15.4	-	-	-	-
	11p15.3	+	FRA11J	-	-
	11q12	-	-	+	-
12	12p13.33	+	FRA12F	-	-
13	13q12.1	+	-	-	-
	13q14	+	FRA13G	?+	-
	13q32 ~ 33	+	FRA13D	+	-
14	14q11.2	+	FRA14D	?+	+
15	15q11.2	+	FRA15C	?+	-
	15q26.1 ~ 26.2	+	FRA15G	?+	-
16	16p13.1	+	FRA16H	+	-
17	17p11.1	-	FRA17C	?+	-
	17q21.3	+	FRA17D	?+	-
	17q24	-	FRA17E	+	-
18	18q21	+	FRA18B	-	-
19	19p13.2	-	-	?+	-
	19q13.2	-	-	+	-
	19q13.43	-	-	?+	-
20	20p12	-	FRA20B	-	-
	20p11.1	-	-	-	-
	20q11.1	-	FRA20D	-	-
21	21q11.2	+	FRA21	?+	-
22	22q11.21	+	-	?+	-
Y	Yp11.31	-	-	-	-
	Yp11.2	+	-	-	-
	Yq11.23	+	-	-	-

*Abbreviations:* - = no; + = yes; ? + = most likely yes; a and b in 5q35.3 = break within subtelomere region.

The centromeric positions could be narrowed down to the subband level for all 24 TCR chromosomes (Table 1). In the following chromosomes the TCR centromeric positions were the same as in HSA: TCR 1 ( = HSA 5), TCR 3 ( = HSA 4), TCR 4 ( = HSA 7), TCR 7 ( = HSA 8), TCR 8 ( = HSA 19), TCR 9 ( = HSA 16), TCR 10 ( = HSA 12), TCR 12 ( = HSA 10), TCR 17 ( = HSA 2), TCR 18 ( = HSA 17), TCR X ( = HSA X), TCR Y1 ( = HSA 5), and TCR Y2 ( = HSA Y). Centromere positions changed compared to HSA in TCR 2 (HSA 3q26), TCR 5 (HSA 15q26.1 ~ 26.2), TCR 6 (HSA 1q22/ 1p14), TCR 11 (HSA 2q24.2), TCR 13 (HSA 11p15.3), TCR 14 (HSA 9q33 ~ 34.1), TCR 15 (HSA 21q11.2/ 22q11.2), TCR 6 (HSA 6q21), TCR 19 (HSA 13q14), TCR 20 (HSA 18q21) and TCR 21 (HSA 20p12).

None of the aforementioned TCR centromeric regions that kept their position during evolution compared to human showed positive FISH-signals with any of the used HSA centromere specific probes (data not shown). Also, none of the other human heterochromatin specific probes from HCM probe set gave any specific signals in TCR, with 2 exceptions: NOR-specific signals were observed in TCR 5 (at fusion of HSA 14q11.2 and 15q26.3) (Figure 1) and the probes midi54 (Figure 1) and midi 36 (data not shown); the latter two located on the distal end of the long arm of TCR 14.

**Figure 2:**
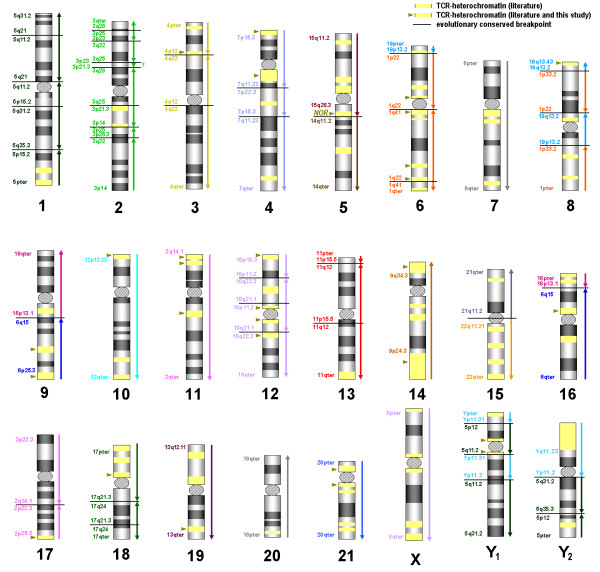
**A summary of the obtained results on TCR idiograms.** The homologous regions of HSA and their orientation are shown as colored arrows on the right of each ideogram. Also the evolutionary conserved breakpoints in respect to HSA nomenclature are inscribed. Consider also legend on top of the figure itself.

In the literature there were 81 TCR specific heterochromatic insertions and/ or additions to chromosomes reported (Figure 2). In this study only 25 of them were confirmed and mapped (Figure 2 and Table 1, last column).

Table 2 summarizes the 69 evolutionary conserved breakpoints observed in TCR in this study; they are given according to the homologous regions in HSA. Only HSA chromosomes X (TCR X) and 8 (TCR 7) are completely unaltered during evolution from a common ancestor of HSA and TCR. All other homologous of TCR chromosomes have undergone one (HSA 12, 14, 16, 18, 21 and 22), two (HSA 4 and 15), three (HSA Y, 11, 13, 17, 19, and 20), four (HSA 1 and 6), five (HSA 2), six (HSA 5, and 10), or nine (HSA 3) evolutionary conserved break events during speciation in respect to the human karyotype.

Besides, the characterized breakpoints of TCR are compared with such previously mapped in *Hylobates lar* (HLA) and *Gorilla gorilla* (GGO) using MCB approach (Table 2). Again, an alignment of the breakpoint and their positioning in GTG-light bands and their spatial proximity to human fragile sites was done.

## Discussion

The present study represents the first one that comprehensively characterizes the karyotype of TCR. In general, previous homologies of HSA and TCR chromosomes could be confirmed [10]. However, in this study, homologous regions for TCR chromosomes 4, 10, 11, 14, 17, 18 and 21 (that were not studied before) [10] were specifically aligned to their HSA-homologous. In contrast to [10] NOR was mapped here to the fusion points of HSA 14 and HSA 15, i.e. TCR 5 and not TCR 15. In our two studied individuals derived from Thailand, no differences in TCR 1 banding pattern were seen, which is in accordance with the literature [10].

For the first time, the exact breakpoints could be determined for the extremely rearranged karyotype of TCR, in comparison to HSA. In fact, 69 evolutionary conserved breakpoints were determined in a male TCR and confirmed in a female individual, excluding Y_1_ and Y_2_ chromosomes, obviously.

In this study no special attention was given to the centromeric regions of TCR, i.e. they were not detailed characterized as in other studies e.g. by [15] or [16]. However, a first impression is provided in which centromeres kept their positions during evolution from common ancestors to HSA and TCR and were neo-centromeres (Table 1), i.e. ~50% of them stayed at the same positions and ~50% moved either in one of the two species or in both. As expected from the literature [17], even the centromeric regions that kept their positions did not have the identical alphoid sequences in HSA and TCR.

Previous studies in human chromosomal rearrangements revealed that the majority of them (70-88%) are found in G-light sub-bands [18]. In contrast only 37 (45.5%) of the 69 evolutionary conserved breakpoints of TCR were located in GTG-light bands (Table 2). However, 70% of the here observed TCR breakpoints colocalized with human fragile sites [19] supporting their potential role in the “Fragile Breakage Model” [20] and in the formation of evolutionary chromosomal rearrangements [21-26] (Table 2).

Concerning evolution it is interesting to report that in TCR and in HLA 11 evolutionary conserved breakpoints are identical and 15 more are most likely in concordance to each other. Even more interesting, 6 identical and 2 most likely identical evolutionary conserved breakpoints were identified in TCR and in GGO (Table 2). These findings need to be confirmed in further studies by locus-specific probes, and if confirmed, they will be very useful for the reconstruction of a common ancestral karyotype. Compared to the postulated Hominidea ancestral karyotype proposed by [25], only four chromosomes remained unchanged in TCR, i.e. chromosomes 4, 7, 11 and X, eleven chromosomes underwent only intrachromosomal changes like inversions (TCR 2, 3, 10, 12, 13, 14, 17, 18, 19, 20, 21) and two TCR chromosomes resulted from a fusion of ancestral chromosomes (TCR 5 and 15).

Interestingly, the regions between TCR 1 and TCR Y_1_ and TCR Y_2_ being homologous to HSA 5 were shown to be subject to different evolutionary conserved rearrangements. Broadly speaking, TCR Y1 is homologous to TCR 1p and TCR Y2 to TCR 1q. However, each arm of chromosome TCR 1 underwent a further paracentric inversion, most likely being important to separate the sex chromosomes from the chromosome 1 during meiosis. Thus, a XY_1_Y_2_ sex chromosome system is present in TCR, and not an X_1_X_2_Y_1_Y_2_ system as initially suggested [10]. However, as in TCR from Indonesia, two other forms of TCR 1 chromosome could be found [10]. Therefore, the existence of an X_1_X_2_Y_1_Y_2_ system cannot be completely excluded by this study.

The sex determination system in mammals is usually highly conserved as XY-system. However, multiple sex chromosome systems, like the one present in TCR and few other apes [27,28] are exceptionally found in some species of e.g. the orders Insectivora, Chiroptera, Artiodactyla, Rodentia [29], and in marsupials [30]. In general, constitutional Y-chromosome / autosome translocations in human appear de novo and have a deleterious effect and, although the infertility is the only common feature, other clinical symptoms can also be observed depending on the involved breakpoints [31]. In such cases, the infertility is thought to be a result of disruption of the sex vesicle during meiosis [32]. From this point of view it is hard to imagine conditions which are in favor of developing a multiple sex- from an XY-chromosome system. On the other hand, in population genetic models of Y-autosome and X-autosome rearrangements the population can gain a selective advantage under a wide range of conditions. If they can invade the population, Y-autosome rearrangements always spread to fixation, whereas X-autosome rearrangements may be maintained as stable polymorphisms” [33]. The XY_1_Y_2_ sex chromosome system observed in TCR fits well into the suggestions of [34] that (i) female meiotic drive is the major contribution to the evolution of neo-sex chromosomes and (ii) that “in mammals, the XY_1_Y_2_ sex chromosome system is more prevalent in species with karyotypes of more biarmed chromosomes” rather than in species with acrocentric chromosomes. Research on meiotic behavior of such sex systems is scarce; however, one study on Bolivian owl monkey (*Aotus spec*.) showed that no XY pairing was observable but the Y-chromosomes formed trivalents with an autosome during gametogenesis [27].

## Conclusions

In conclusion, the presented comparative map of TCR karyotype gives new insights into primate evolution and can be used as a starting point for further detailed analyses. Evolutionary conserved breakpoints, TCR-specific heterochromatic regions, centromeric sequences as well as the sex chromosome system can be fruitful fields of research in near future.

## Methods

### Cell culture and chromosomal preparation

Immortalized lymphoblast cell lines derived from a male and female TCR, were provided by the Khon Kaen University, Thailand. Culture techniques and chromosome preparation followed standard protocols.

### Fluorescence in situ hybridization

General considerations for MCB labeling schemes and details regarding probe preparation and labeling have been described before [12,35,36]. Single and dual-color FISH techniques were performed for the applied bacterial artificial chromosome (BAC-) probes [37]. Locus-specific BAC clones were purchased at BAC/PAC Chori and DNA was isolated, PCR-amplified and labeled as described before [37]; and probes RP11-475I16 and RP11-395 L14 (both in 2q14.1), RP11-110A24 (in 19p13.3), RP-11457 M7 (in Yp11.2) and RP11-122 L9 (in Yp11.31) were applied in this study. Besides, commercially available human derived probes for the SRY gene on the Y-chromosome, HSA probes for subtelomeric regions 3pter, 3qter, 5pter, 5qter, 6pter, 6qter, 7pter, 7qter, 9pter, 9qter, 10pter, 10qter, 18pter, 18qter, 19pter, 19qter, XYpter and XYqter, and centromeric probes cep 2, cep4, cep 7, cep 8, cep 10, cep 12, cep 16, cep 17, cep X cep Y (Abbott/ Vysis, Wiesbaden, Germany) and SE 1/5/19, SE 13/21 and SE 14/22 (Kreatech, Amsterdam, The Netherlands) were also applied.

Additionally, the following homemade HSA derived microdissection probes were used: a probe specific for the short arm of all human acrocentric chromosomes (midi54) [12,13], and partial chromosome paints for 3p, 3q, 5p, 5q, 6p, 6q, 7p, 7q, 9p, 9q, 10p, 10q, 18p, 18q, 19p, 19q, Yp and Yq [35]. Furthermore, the heterochromatin mix (HCM-) probe set [38] covering chromosomal regions 1q12, 16q11.2, 9q12, 9p12/ 9q13 (midi36) 15p11.2-p11.1, 19p12/q12 and Yq12 and subcentromere specific multicolor FISH (subcen-FISH) for chromosomes 3, 6, 7, 9, 11, 13 and 20 were also applied [36].

### Microscopic evaluation

Images were captured using an Axioplan II microscope (Carl Zeiss Jena GmbH, Germany) equipped with filter sets for DAPI, FITC, TR, SO, Cy5 and DEAC. Image analysis was done using pseudocolor banding and fluorochrome profiles of the ISIS digital FISH imaging system (Meta Systems Hard & Software GmbH, Altlussheim, Germany). At least 10 up to 20 metaphases were recorded, derived from a male and a female TCR for each applied probe and probe set.

## Competing interests

Authors declare that there is no conflict of interest.

## Authors’ contributions

XF carried out molecular cytogenetic studies and the draft of the manuscript, KP carried out the molecular cytogenetic studies, HM carried out the molecular cytogenetic studies, PS carried out the molecular cytogenetic studies, NK carried out the molecular cytogenetic studies, WS carried out the cell culture, AT carried out the cell culture, AC carried out the cell culture and participated in manuscript discussion, TL participated in the design of the study and the manuscript, MBC participated in the design of the study and the manuscript discussion, AW participated in the design and coordinated the study and the manuscript. All authors read and approved the final manuscript.
